# Flurbiprofen inhibits cell proliferation in thyroid cancer through interrupting HIP1R-induced endocytosis of PTEN

**DOI:** 10.1186/s40001-022-00658-3

**Published:** 2022-02-24

**Authors:** Nengli Yang, Yafeng Liang, Pei Yang, Liuming Jiang

**Affiliations:** 1 Department of Anesthesiology, The First Affiliated Hospital of Wenzhou Medical University, 2 Fuxue Street, Wenzhou, 325000 Zhejiang People’s Republic of China; 2 Department of Pediatric Intensive Care Unit, The Second Affiliated Hospital & Yuying Children’s Hospital, Wenzhou Medical University, Wenzhou, 325000 Zhejiang People’s Republic of China

**Keywords:** Thyroid cancer, Endocytosis, HIP1R, PTEN, Flurbiprofen

## Abstract

**Background:**

The incidence of thyroid cancer, a most common tumor in the endocrine system, has increased in recent years. A growing number of studies have focused on the molecular mechanisms of thyroid cancer subtypes, aiming to identify effective therapeutic targets. Endocytosis is of vital significance in the malignant development of tumors, although its involvement in thyroid cancer has been rarely reported.

**Methods:**

HIP1R expressions in thyroid cancer from the TCGA database were analyzed by UALCAN software. Thyroid epithelial and cancer cell lines were cultured in vitro. Western blotting and quantitative PCR were used to analyze protein and mRNA levels, respectively. Cell viability was measured by CCK-8 assay. Immunofluorescence staining indicated protein distribution in cell. Co-immunoprecipitation was used to study protein–protein interaction. Immunohistochemical staining was used to analyze protein expression in clinical tissues. Differences between groups were compared using the two-tailed Student’s *t* test, and those among three or more groups were compared by one-way or two-way ANOVA.

**Results:**

In the present study, HIP1R (Huntingtin Interacting Protein 1 Related) was found upregulated in thyroid cancer tissues and cell lines compared with that in the controls, while knockdown of HIP1R significantly inhibited the proliferation of thyroid cancer cells. Since HIP1R is essential for the clathrin-dependent endocytic process, we thereafter explored the effect of HIP1R on the endocytosis of thyroid cancer cells. Interestingly, knockdown of HIP1R significantly reduced the number of clathrin-coated pits (CCPs) in thyroid cancer cells. In addition, the interaction between HIP1R and PTEN (phosphatase and tensin homolog) was identified in thyroid cancer cells. Knockdown of HIP1R downregulated intracellular PTEN in thyroid cancer cells, but upregulated membrane-binding PTEN. Notably, flurbiprofen, a commonly used analgesic, significantly inhibited the proliferation of thyroid cancer cells and interfered with the interaction between HIP1R and PTEN, thereby enhancing the binding of PTEN to cell membrane. However, the proliferation inhibitory effect of flurbiprofen was attenuated when knocking down HIP1R or PTEN.

**Conclusions:**

Upregulated HIP1R in thyroid cancer cells promotes cell proliferation and mediates the endocytosis of PTEN. Flurbiprofen may exert an anti-tumor effect on thyroid cancer by blocking the interaction between HIP1R and PTEN.

## Introduction

Thyroid cancer, a common endocrine system tumor [1], mostly originates from thyroid follicular cells (> 90%). Thyroid cancer is histologically classified into well-differentiated papillary thyroid cancer (PTC, 90%), follicular thyroid cancer (FTC) and medullary thyroid cancer (MTC, less than 5%) [2]. Among them, PTC and FTC may develop into poorly differentiated thyroid cancer (PDTC) or anaplastic thyroid cancer (ATC) [3]. Although most thyroid cancers are benign hyperplastic tumors, some PDTC patients die from the long-term progression of the tumor, with a 5-year disease-specific survival (PSF) of 66% [4]. Therefore, it is of great significance to explore the molecular mechanism underlying the pathogenesis of thyroid cancer, especially PDTC and ATC.

During endocytosis, cell surface proteins (e.g., cytokine receptors and adhesion components) are selectively packaged in cytoplasmic vesicles (endosomes), and subsequently, internalized proteins are either degraded in the lysosome or recycled back to the plasma membrane [5]. In tumor cells, abnormal endocytosis is found, which is closely linked with the uncontrolled proliferation, metastasis, and anti-apoptosis of malignant phenotypes [6, 7]. HIP1R, an endocytic adaptor protein of the Sla2/HIP1 family [8], contains an ANTH domain binding to inositol phospholipids, a coiled domain binding to the light chain of clathrin, and a THATCH domain binding to F-actin [9]. HIP1R is also capable of binding to other endocytic proteins like Cortactin [10], Epsin [11] and CIN85 [12]. It is reported that Sla2p, the yeast homologue of HIP1R, is essential for endocytosis and functional actin cytoskeleton [13]. Consistently, HIP1R in mammalian cells is responsible for clathrin-mediated endocytosis and actin assembly [10]. The role of HIP1R and HIP1R-induced endocytosis in thyroid cancer, however, has not been reported.

Inflammation is strongly associated with cancer and plays a crucial role in tumor development and progression. It is well known that chronic inflammation promotes carcinogenesis by inducing proliferation, angiogenesis, metastasis and therapeutic resistance [14]. Non-steroidal anti-inflammatory drug (NSAID) is one of the most widely used analgesic and anti-inflammatory drug groups in the world. However, the anticancer effects of NSAIDs have recently been a remarkable topic, because mortality was significantly reduced in cancer patients after combination therapy with NSAIDs [15]. Compared with other NSAIDs, flurbiprofen has strong efficacy, rapid onset, long duration and few adverse reactions, which is also associated with longer survival in patients who underwent tumor surgery [16, 17]. Although flurbiprofen has been applied as an analgesic in thyroid surgery [18, 19], whether it has anticancer activities against thyroid cancer has not been explored.

Based on the present study, we hypothesized that the upregulated HIP1R in thyroid cancer cells promoted cell proliferation through mediating endocytosis of PTEN, and flurbiprofen, a highly effective nonsteroidal anti-inflammatory drug (NSAID) inhibited the endocytosis by interrupting the interaction between HIP1R and PTEN, consequently attenuating the proliferation of thyroid cancer cells. Therefore, our findings provide a novel experimental and theoretical basis for the treatment of thyroid cancer.

## Materials and methods

### Overview

Firstly, we analyzed the mRNA levels of HIP1R in thyroid cancer based on the TCGA database by UALCAN software. Then, we measured and compared the protein levels of HIP1R in thyroid cancer and adjacent tissues by immunohistochemical staining. Next, western blotting and quantitative PCR were used to analyze protein and mRNA levels of HIP1R in in vitro cultured thyroid cancer cells. Secondly, in the phenotype experiments, the effect of HIP1R on the viability of thyroid cancer cells was examined by CCK-8 assay. Thirdly, in the experiments for mechanism, detecting the signal of HIP1R-GFP or clathrin-GFP by fluorescence microscope indicated the formation of endosome. Furthermore, co-immunoprecipitation was used to study the interaction between HIP1R and PTEN, while immunofluorescence staining revealed the effect of HIP1R on the distribution of PTEN protein in thyroid cancer cells.

### Cell culture and treatment

The human normal thyroid epithelial cell line NTHY-ORI 3–1, human papillary thyroid cancer cell line TPC-1, human poorly differentiated thyroid cancer cell line SW579, and human undifferentiated thyroid cancer cell line 8505C were provided by the American Type Culture Collection (ATCC; Manassas, VA, USA). NTHY-ORI 3–1 and TPC-1 cells were cultured in RPMI-1640 containing 10% fetal bovine serum (FBS, Gibco, Carlsbad, CA); SW579 cells were cultured in Leibovitz’s L-15 medium containing 10% FBS; 8505C cells were cultured in DMEM containing 10% FBS. All cells were cultured in a 5% CO_2_ incubator at 37 °C. Cells were treated with 10 nM flurbiprofen (referring to the half-maximal inhibitory concentration of flurbiprofen in colorectal cancer cells [20]) or 0.1% DMSO (as the negative control).

### Plasmids, siRNAs and transfection

HIP1R-GFP and clathrin-GFP were constructed using the pcDNA3.1 vector. Control, HIP1R and PTEN siRNAs (siCtrl, 5’-UUCUCCGAACGUGUCACGUTT-3ʹ; siHIP1R, 5ʹ-GGAUUGUGAGCUGAAGCUUUCUGAATT-3ʹ; siPTEN, 5ʹ-UUCCAUUUUCAAUAACUUAUUGGTT-3ʹ) were synthesized by GenePharma (Shanghai, China). The overexpression and knockdown were performed by transfection with plasmids and siRNAs, respectively, using the Lipofectamine™ 3000 (Invitrogen; ThermoFisher Scientific, Waltham, MA, USA) following the manufacturer’s protocol (Publication No. MAN0009872).

### Immunohistochemical staining (IHC)

A total of 28 pairs of thyroid cancer and adjacent normal tissues were provided by the National Infrastructure of Chinese Genetic Resources (NICGR, 2005DKA21300), all with written informed consent. The study was approved by the ethics committee of the First Affiliated Hospital of Wenzhou Medical University. Using the EnVision system (Dako, Carpinteria, CA, USA), 100 cells were counted in each of 8 randomly selected fields per sample at the magnification of 200×, and the IHC intensity was graded into 0, no reaction; 1, weak staining; 2, mild staining; and 3, strong staining. The ratio of IHC-stained cells was graded into 0, < 5%; 1, 6–25%; 2, 26–50%; 3, 51–75%; and 4, > 75%. IHC score was finally calculated by multiplying the IHC intensity grade and IHC-stained cell grade, in which a low expression and high expression were defined at 0–6 and 7–12 scores, respectively. IHC results were independently determined by two investigators blinded to clinical data of subjects, and any disagreement was solved by discussion.

### Western blot

Cells were lysed in RIPA (Millipore, Temecula, CA) containing phenylmethanesulfonyl fluoride or phenylmethylsulfonyl fluoride (PMSF) for collecting total proteins, and their concentrations were measured using the Bradford method (Bio-Rad Laboratories Inc., Hercules, CA, USA). Protein samples were loaded 40 μg per lane for SDS-PAGE, and transferred on the nitrocellulose membrane. After blocking in TBST containing 5% skim milk at room temperature for 2 h, it was incubated with anti-HIP1R (ab140608, Abcam, Cambridge, UK), anti-PTEN (sc-7974, Santa Cruz, Dallas, TX, USA) and anti-GAPDH (sc-365062, Santa Cruz, 1: 5 000) antibodies at 4 ℃ overnight. After TBST wash, it was incubated with IRDye 800CW goat anti-rabbit IgG (H + L) secondary antibody (Cat.No.926-32211) and IRDye 800CW goat anti-mouse IgG (H + L) secondary antibody (CAT.No.926-32210, 1:10 000) at room temperature for 1 h. Band exposure was achieved using the Odyssey imaging system (Li-Cor Biosciences, Lincoln, NE, USA) and transformed to grey values using the Application Software (version 2.1.12, Li-Cor Biosciences).

### Quantitative real-time PCR (qRT-PCR)

Total RNA was extracted using RNAisoTM Plus (Takara, OTSU, Japan) and the concentration was measured using a NanoDrop ND-1000 spectrophotometer. After reverse transcription of 0.5 μg RNA using the PrimeScript™ RT kit (Takara), qRT-PCR was performed on the Step One Plus Real-Time PCR system (Thermo Fisher Scientific Inc.) using the SYBR-Green Master Mix (Roche Diagnostics, Basel, Switzerland) at 95 ºC for 5 min, followed by 40 cycles at 95 ºC for 45 s, 55 ºC at 45 s and 72 ºC for 1 min. Relative level was calculated by the 2^−ΔΔCt^ method and normalized to that of β-actin. Primer sequences were as follows: HIP1R, 5ʹ-GCAGGATGAACAGCATCAAGA-3ʹ (forward) and 5ʹ-CCAATGGCGTAGGACCAGAA-3ʹ (reverse); β-actin, 5ʹ-CTCCATCCTGGCCTCGCTGT-3ʹ (forward) and 5ʹ-GCTGTCACCTTCACCGTTCC-3ʹ (reverse).

### CCK-8 assay

Cell viability was measured by CCK-8 assay (Cell Counting Kit-8, Dojindo, Kumamoto, Japan). Briefly, 5 × 10^3^ cells per well were seeded in a 96-well plate. After cell culture for different time points, fresh medium containing 10% CCK-8 solution was added in each well and incubated at 37 ºC for 2 h. Then optical density (OD) at 450 nm wavelength was measured using a microplate reader.

### Co-immunoprecipitation (co-IP)

1 × 10^7^ cells were lysed in RIPA (Millipore) to collect total protein, and the concentration was measured using the Bradford method (Bio-Rad). 0.5–1 mg total protein was incubated with 1 μg recombinant anti-HIP1R antibody (ab140608, Abcam) in 0.5 ml of PBS at 4 ºC for 16 h, followed by incubation with 25 μl of protein A/G at 4 ºC for another 2 h. Immunoprecipitation of PTEN in anti-IgG and anti-HIP1R was finally determined by Western blot.

### Immunofluorescence staining

5 × 10^4^ cells were seeded in each well of a 6-well plate and transfected at 24 h. They were fixed in 4% pre-cold paraformaldehyde after 48-h transfection. Following permeabilization in 0.2% Triton X-100 and blockage of non-specific antigens in 20% BSA, cells were incubated with the anti-PTEN antibody (sc-7974, Santa Cruz) at 4 ºC overnight. After PBS wash, cells were incubated with the goat anti-mouse IgG (H + L) secondary antibody Alexa Fluor®488 conjugate (#4408, CST, 1:500) at room temperature in the dark. Cell nuclei were stained with DAPI in the dark. Immunofluorescence staining images were captured using a fluorescence microscope (Axio Observer D1, Zeiss, Göttingen, Germany).

### Statistical analysis

Statistical analysis was conducted using SPSS 22.0 (IBM Corp., Armonk, NY, USA) and GraphPad Prism 5.0 (GraphPad Software Inc., La Jolla, CA, USA). Data were expressed as mean ± standard deviation from three independent experiments. Differences between groups were compared using the two-tailed Student’s *t* test, and those among three or more groups were compared by one-way or two-way ANOVA, followed by Turkey’s range test or Šidák correction, respectively. *P* < 0.05 was considered as statistically significant.

## Results

### HIP1R is upregulated in thyroid cancer tissues

**Fig. 1:**
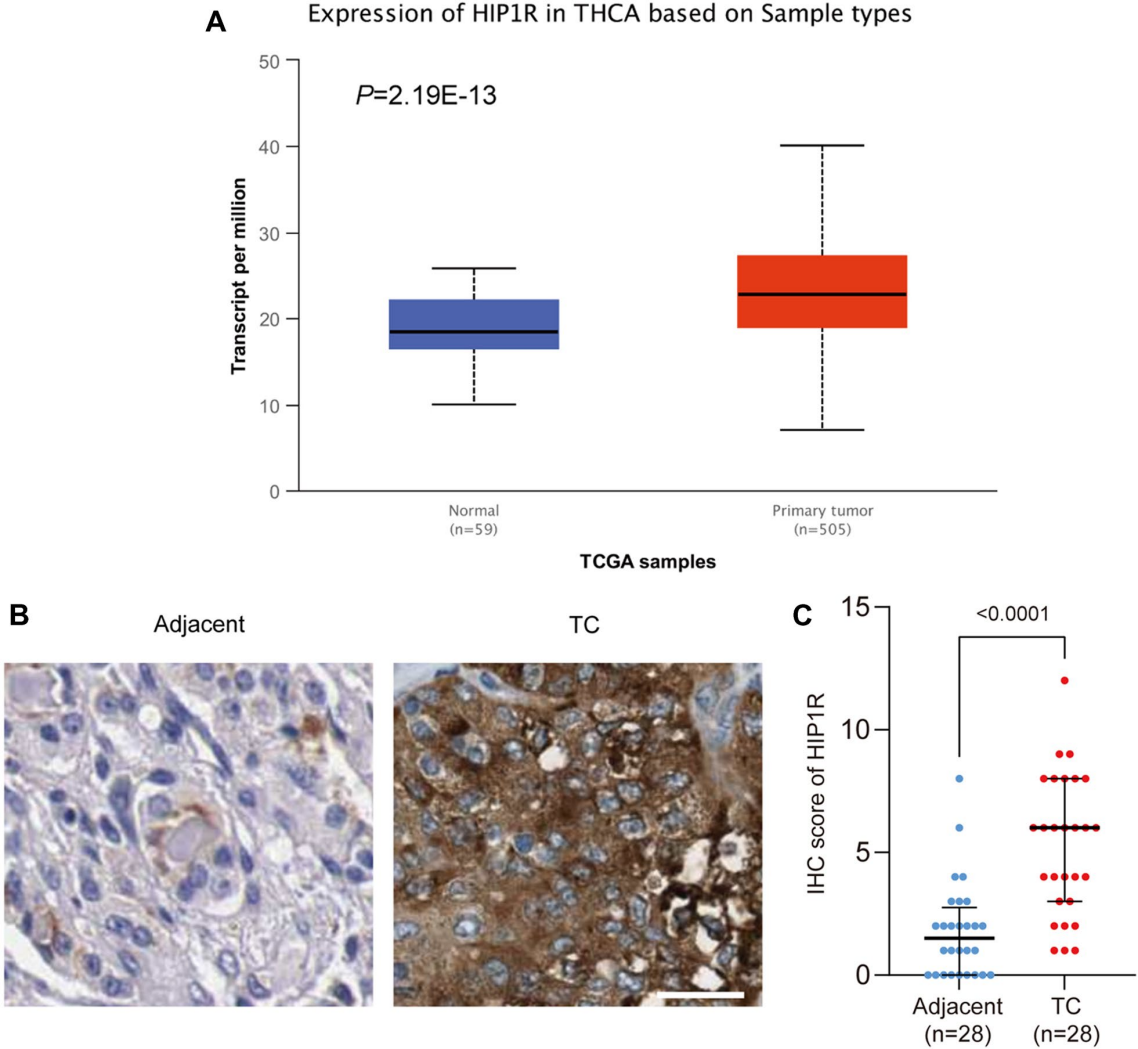
HIP1R is upregulated in thyroid cancer tissues. **A** The mRNA level of HIP1R in the dataset containing thyroid cancer tissues (*n* = 505) and normal thyroid tissues (*n *= 59) in the TCGA database analyzed using the online tool UALCAN. **B** Representative images of IHC staining of HIP1R in thyroid cancer tissues and normal ones. Scale bar = 100 μm. **C** IHC score of HIP1R in thyroid cancer tissues (*n* = 28) and paracancerous ones (*n* = 28)

The expression level of HIP1R in the dataset containing thyroid cancer tissues and normal ones in the TCGA database was analyzed using the online tool UALCAN (http://ualcan.path.uab.edu/analysis.html), which was found significantly upregulated in thyroid cancer tissues compared with that in normal tissues (Fig. 1A). Moreover, the IHC score of HIP1R was significantly higher in thyroid cancer tissues than that in normal tissues (Fig. 1B and C).

### Knockdown of HIP1R inhibits the proliferation of thyroid cancer cells

**Fig. 2:**
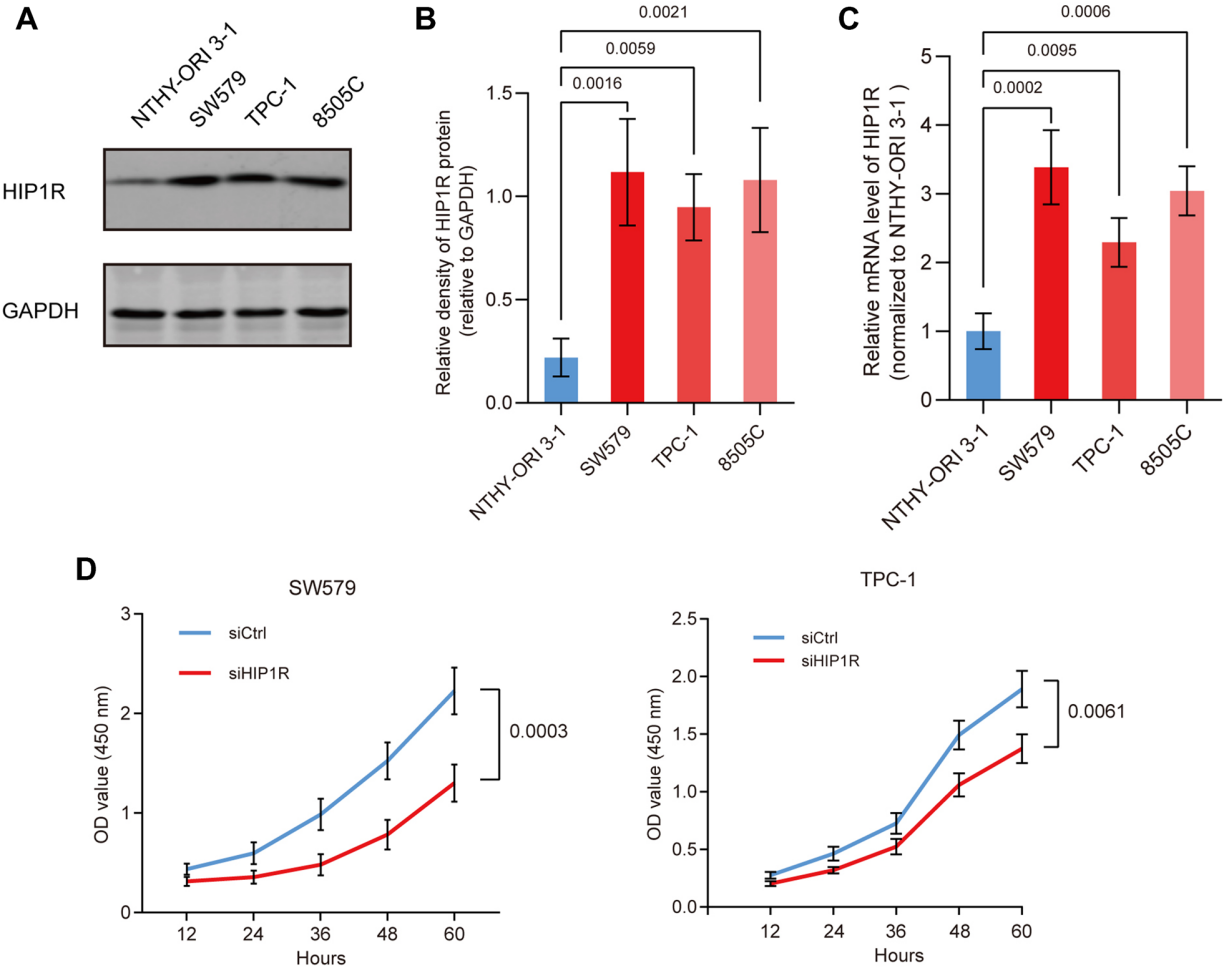
Knockdown of HIP1R inhibits the proliferation of thyroid cancer cells. **A** Protein level of HIP1R in NTHY-ORI 3–1, SW579, TPC-1 and 8505C cells examined by Western blot. GAPDH was the internal reference. **B** Grey value of HIP1R normalized to that of GAPDH calculated by Image J. **C** The mRNA level of HIP1R in NTHY-ORI 3–1, SW579, TPC-1 and 8505C cells examined by qRT-PCR. β-actin was the internal control. **D** Cell viability at 12, 24, 36, 48 and 60 h in SW579 and TPC-1 cells transfected with siCtrl or siHIP1R, respectively, examined by CCK-8 assay

To further explore the potential function of HIP1R in the development of thyroid cancer, we measured its expression level in thyroid cancer cell lines. Compared with the human normal thyroid epithelial cell line NTHY-ORI 3–1, mRNA and protein levels of HIP1R were both significantly upregulated in human papillary thyroid cancer cell line TPC-1, human poorly differentiated thyroid cancer cell line SW579, and human undifferentiated thyroid cancer cell line 8505C (Fig. 2A–C). CCK-8 assay revealed that knockdown of HIP1R significantly reduced the viability in SW579 and TPC-1 cells, indicating the inhibited proliferation (Fig. 2D). It is concluded that HIP1R is of significance in the proliferation of both poorly differentiated and papillary thyroid cancer cells.

### HIP1R promotes clathrin-dependent endocytosis in thyroid cancer cells

**Fig. 3:**
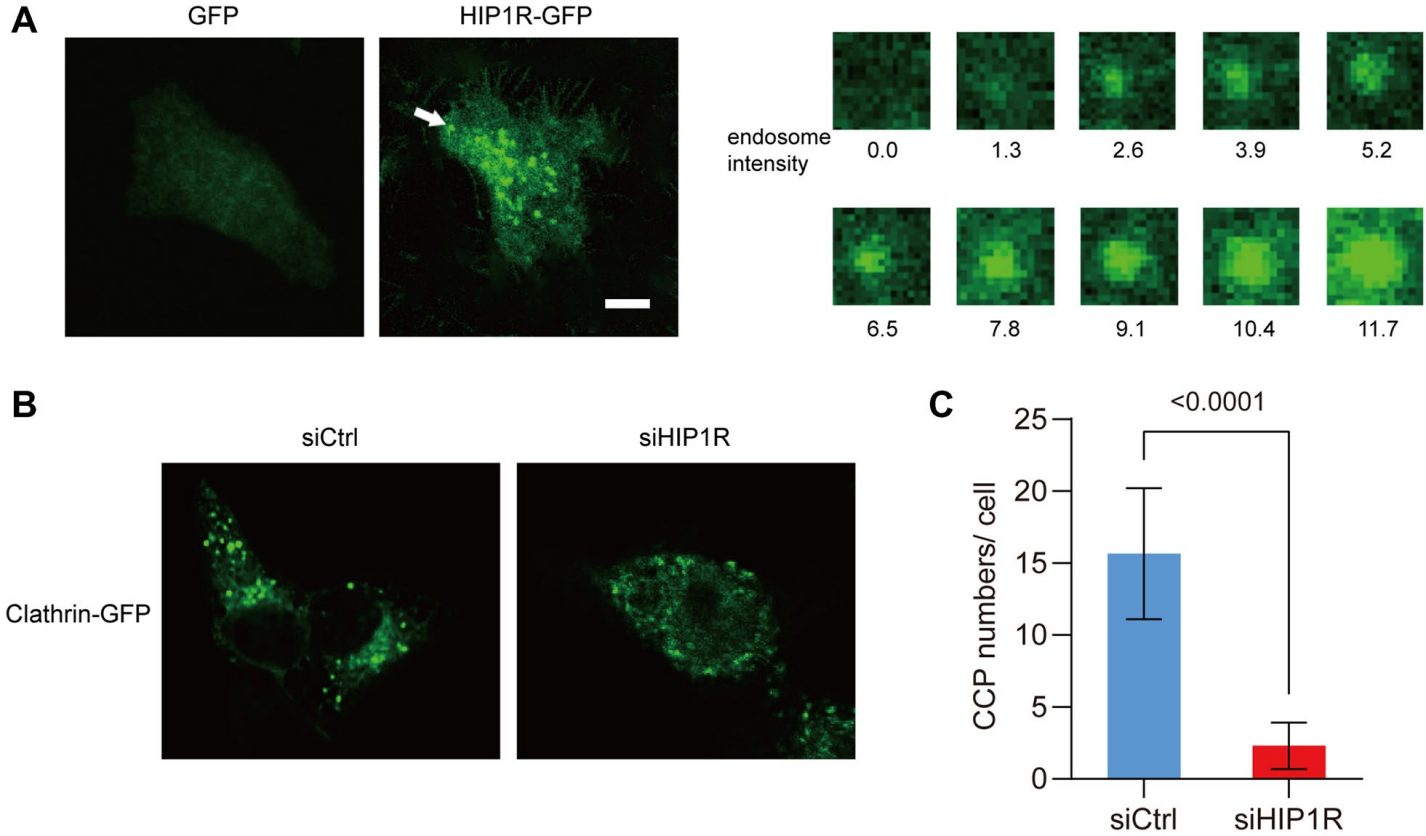
HIP1R promotes clathrin-dependent endocytosis in thyroid cancer cells. **A** The formation of endosomes (white arrow) in HIP1R-GFP-labeled SW579 cells. Scale bar: 10 μm. **B** Clathrin-GFP-labeled CCPs in SW579 cells transfected with siCtrl or siHIP1R for 48 h, respectively. Scale bar: 10 μm. **C** The mean number of CCPs in each cell of 20 SW579 cells with HIP1R knockdown

Previous evidence has shown the vital function of HIP1R in cell endocytosis. Here, SW579 cells were labeled with HIP1R-GFP for overexpression of HIP1R, followed by observation of endosome formation by immunofluorescence staining. As shown in Fig. 3A, the formation of endosomes was significantly enhanced in SW579 cells overexpressing HIP1R (Fig. 3A). On the contrary, knockdown of HIP1R significantly reduced the number of CPPs in SW579 cells (Fig. 3B and C). It is suggested that highly expressed HIP1R promotes clathrin-dependent endocytosis in thyroid cancer cells.

### HIP1R induces endocytosis of PTEN in thyroid cancer cells

**Fig. 4:**
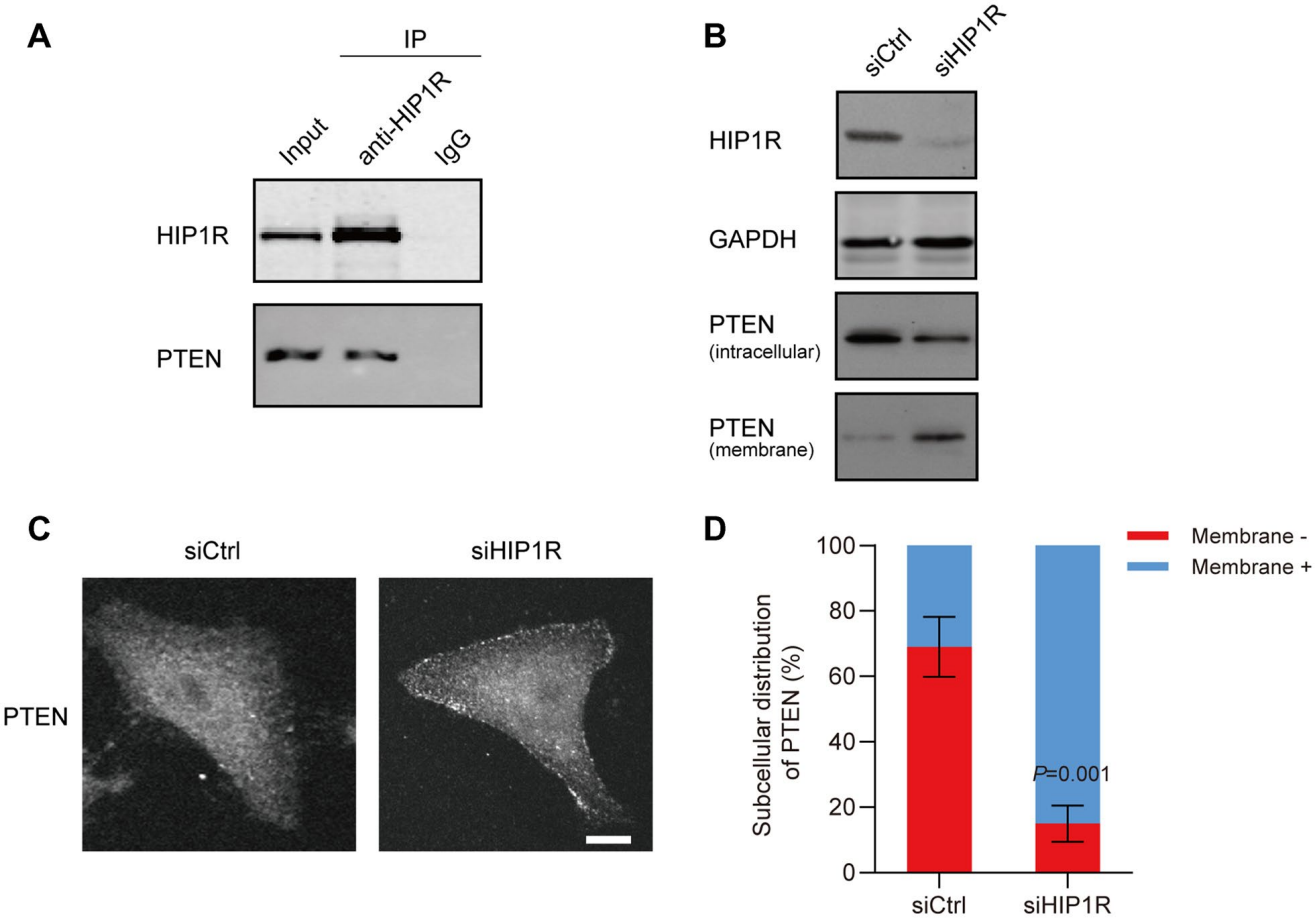
HIP1R induces endocytosis of PTEN in thyroid cancer cells. **A** The interaction between HIP1R and PTEN in SW579 cells examined by co-IP. **B**–**D**: SW579 cells were transfected with siCtrl or siHIP1R for 48 h, respectively. **B** Protein levels of membrane-binding PTEN and intracellular PTEN in SW579 cells. GAPDH was the internal control. **C** Fluorescence staining of PTEN in SW579 cells. Scale bar: 10 μm. **D** Subcellular distribution ratio of intracellular PTEN and membrane-binding PTEN in 100 SW579 cells

It is reported that PTEN can inhibit the proliferation of thyroid cancer cells [21], and the biological function of PTEN is influenced by endocytosis [22]. We speculated that the endocytosis of PTEN in thyroid cancer cells might be specifically induced by HIP1R. An interaction between PTEN and HIP1R in SW579 cells was first validated by co-IP assay (Fig. 4A). Importantly, knockdown of HIP1R in SW579 cells upregulated membrane-binding PTEN, but downregulated intracellular PTEN (Fig. 4B). The ratio of SW579 cells with positive staining of membrane-binding PTEN, as expected, was significantly increased by knockdown of HIP1R (Fig. 4C and D). It is suggested that highly expressed HIP1R may promote the endocytosis of PTEN in thyroid cancer.

### Flurbiprofen inhibits proliferation of thyroid cancer cells by affecting the HIP1R-mediated endocytosis of PTEN

**Fig. 5:**
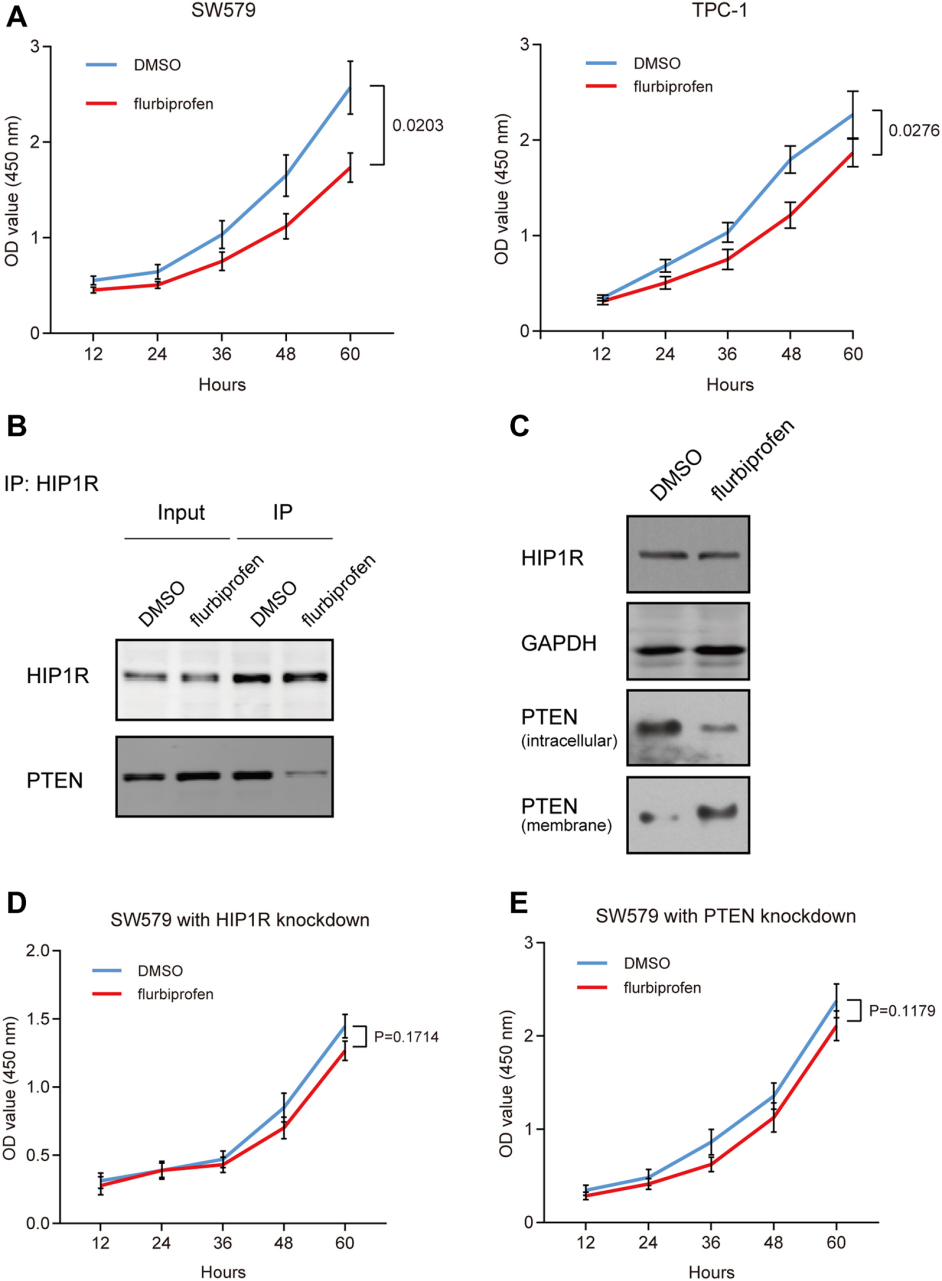
Flurbiprofen inhibits proliferation of thyroid cancer cells and affects HIP1R-mediated endocytosis of PTEN. SW579 and TPC-1 cells were treated with 10 nM flurbiprofen or DMSO. **A** Cell viability at 12, 24, 36, 48 and 60 h in SW579 and TPC-1 cells examined by CCK-8 assay. **B** After treatment with flurbiprofen for 48 h, the interaction between HIP1R and PTEN in SW579 cells examined by co-IP. **C** After treatment with flurbiprofen for 48 h, protein levels of membrane-binding PTEN and intracellular PTEN in SW579 cells. GAPDH was the internal control. **D** and **E** SW579 cells were transfected with siCtrl, siHIP1R or siPTEN and also treated with 10 nM flurbiprofen or DMSO. Cell viability at 12, 24, 36, 48 and 60 h in SW579 cells examined by CCK-8 assay

Flurbiprofen is a commonly used NSAID to inhibit the growth of multiple tumors [16]. Our findings consistently revealed that flurbiprofen treatment effectively decreased the viability of SW579 and TPC-1 cells (Fig. 5A). Interestingly, the interaction between HIP1R and PTEN was interfered by flurbiprofen (Fig. 5B). Similar to the outcomes of HIP1R knockdown, flurbiprofen treatment resulted in the upregulation of membrane-binding PTEN and downregulation of intracellular PTEN (Fig. 5C). However, flurbiprofen could not suppress the proliferation of SW579 cells with knockdown of HIP1R or PTEN (Fig. 5D and E). Based on the above findings, we hypothesize that flurbiprofen may inhibit the proliferation of thyroid cancer cells via affecting HIP1R-mediated endocytosis of PTEN.

## Discussion

HIP1R is capable of mediating cell growth and survival [23]. It is also involved in tumor development. In prostate cancer, overexpression of HIP1R triggers cell migration, invasion and non-anchored growth [24]. Non-small cell lung cancer (NSCLC) patients with a high HIP1R level suffer from a worse progression-free survival (PFS) than those with a low HIP1R level [25]. The tumor-suppressing effect of HIP1R has also been reported. HIP1R induces apoptosis of gastric cancer cells, and inhibits cell proliferation, invasion, and migration [26]. Through binding to PD-L1, HIP1R delivers PD-L1 to lysosomes via endocytosis, thereby enhancing T cell-dependent cytotoxicity in colorectal cancer [27]. In the present study, HIP1R was upregulated in thyroid cancer tissues and cell lines. Knockdown of HIP1R significantly inhibited proliferation of thyroid cancer cells and clathrin-dependent endocytosis. However, how HIP1R promoted proliferation of thyroid cancer remains unclear. Based on the previous findings, we speculated that HIP1R may alter the downstream signaling pathways of cells/growth factor receptors by promoting their endocytosis, thus stimulating the proliferative capacity of tumor cells.

Phosphatase and tensin homolog (PTEN) has been recognized as an important tumor-suppressor gene, which antagonizes the phosphatidylinositol 3-kinase/protein kinase B (PI3K–AKT) signaling pathway in human tumors [28]. A growing number of studies have shown the involvement of dysregulated PTEN in the malignant development of multiple types of tumors, including thyroid cancer [29]. Mutations and abnormal expression of PTEN ultimately trigger the survival, growth, proliferation, and anti-apoptosis features of tumor cells. Loss of PTEN expression occurs in 10% of thyroid cancers, which is more commonly detected in PDTC and ATC [30, 31]. Promoter methylation of the PTEN gene can be detected in more than 50% of thyroid cancer cases [32]. In addition, abnormally expressed miRNAs and lncRNAs in thyroid cancer cells also result in PTEN dysregulation [21, 33]. Our results proved the interaction between HIP1R and PTEN in thyroid cancer cells, which further downregulated membrane-binding PTEN via inducing the endocytosis of PTEN. The escape from the local high concentration of phosphatidylinositol (3, 4, 5)-trisphosphate [PI[3, 4, 5]P3] of receptor tyrosine kinase (RTK) results in the decline of the antagonistic effect of PTEN on the AKT signaling pathway [28]. Therefore, we speculated that overexpression of HIP1R might promote proliferation signal of thyroid cancer cells by inducing the endocytosis of PTEN.

Flurbiprofen is a highly effective NSAID used extensively in preemptive analgesia, postoperative analgesia, and cancer pain alleviation [34]. Apart from its analgesic effects, flurbiprofen also presents a broad-spectrum anti-tumor effect. It is reported that flurbiprofen is capable of inhibiting cell proliferation in tumors, including colon cancer, gastric cancer, cervical cancer, and liver cancer [20, 35, 36]. It exerts an anti-proliferation effect in tumor cells via non-specifically inhibiting cyclooxygenase 2 (COX2) [37]. Besides, it can suppress tumor angiogenesis by inactivating vascular endothelial growth factors and fibroblast growth factors [38]. Flurbiprofen also stimulates apoptosis of tumor cells, and even enhances therapeutic efficacy of tumor immunotherapy [39, 40]. Our study found that flurbiprofen interfered with the interaction between HIP1R and PTEN, thereby inhibiting proliferation of thyroid cancer cells via blocking the endocytosis of PTEN. The exact molecular mechanism underlying the binding of HIP1R to PTEN, however, is still unclear and needs further explorations.

There are several limitations of the present study. The mechanism that flurbiprofen disturbs the interaction between HIP1R and PTEN in thyroid cancer cells has not been explored. Besides, PTEN has been found to bridge PI3K signaling and endocytosis, as reduction or loss of PTEN promotes the formation of PI3K signaling-induced clathrin-coated pits [41, 42]. So, it is worth determining whether HIP1R-involved endocytosis also depends on the reduction of membrane-binding PTEN, which, in turn, further decreases the level of membrane-binding PTEN in thyroid cancer.

**Fig. 6:**
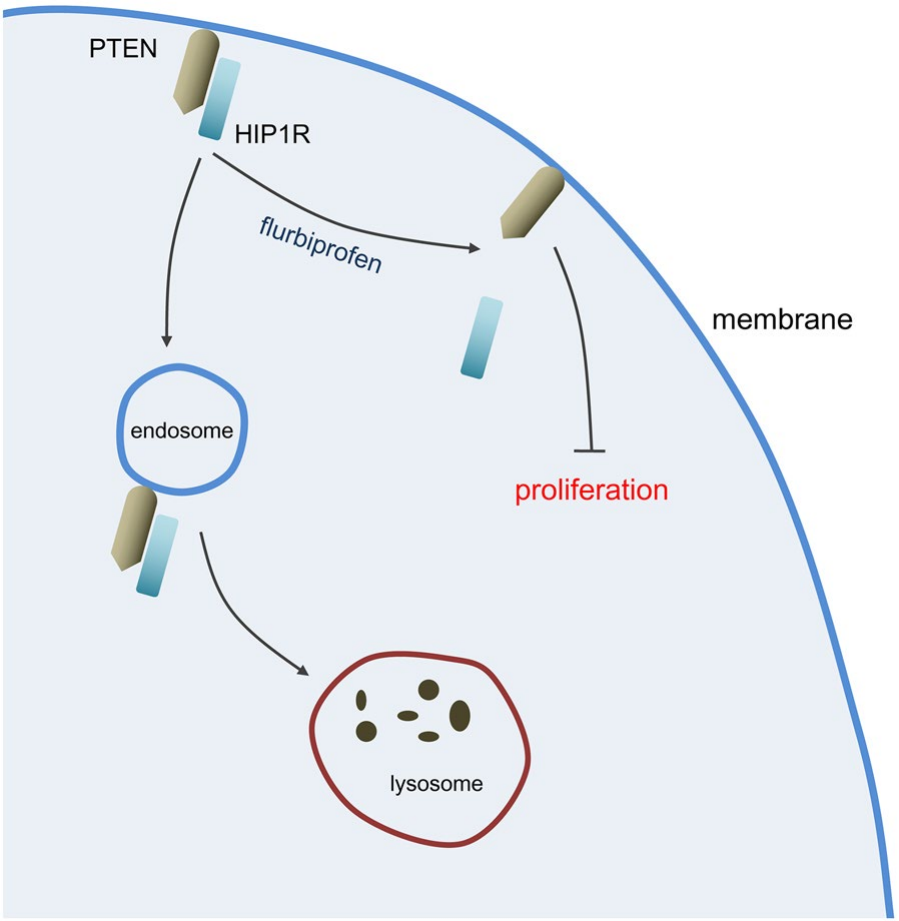
Mechanism scheme underlying the inhibitory effect of flurbiprofen on proliferation of thyroid cancer cells via affecting HIP1R-mediated endocytosis of PTEN. HIP1R is upregulated in thyroid cancer cells, which interacts with PTEN and further induces clathrin-dependent endocytosis of PTEN. As a result, the anti-proliferation role of PTEN in thyroid cancer is damaged. Flurbiprofen inhibits proliferation of thyroid cancer cells by interfering with the interaction between HIP1R and PTEN, thus blocking the endocytosis of PTEN

Combined with the previous reports and present findings, we speculate that upregulated HIP1R promotes thyroid cancer cell proliferation by interacting with PTEN and further inducing clathrin-dependent endocytosis of PTEN. Flurbiprofen inhibits proliferation of thyroid cancer cells by interfering with the interaction between HIP1R and PTEN, thus blocking the endocytosis of PTEN (Fig. 6). Therefore, flurbiprofen may serve as a potential therapeutic drug for thyroid cancer, not just an anesthetic, which has a promise to be used in thyroid cancer surgery.

## Data Availability

All of the data and materials are available.
